# Impact of COVID-19 on the epidemiology of severe sinogenic and otogenic infections and their intracranial complications

**DOI:** 10.1007/s00431-025-06188-4

**Published:** 2025-05-24

**Authors:** Dimitra Dimopoulou, Maria M. Berikopoulou, Ioannis Tsoliakos, Athanasios Michos

**Affiliations:** 1 2nd Department of Pediatrics, ”Aghia Sofia” Children’s Hospital, Athens, Greece; 2 Division of Infectious Diseases, 1st Department of Pediatrics, National and Kapodistrian University of Athens, Thivon and Papadiamantopoulou, 11527 Athens, Greece

**Keywords:** Mastoiditis, Orbital cellulitis, Children, COVID-19, Intracranial infections, Empyema, Sinogenic infections, Otogenic infections

## Abstract

This study aims to evaluate the impact of COVID-19 pandemic on the incidence, microbiological characteristics, management, and outcomes of mastoiditis and orbital cellulitis and their intracranial complications in pediatric patients. A retrospective observational study was conducted at the major pediatric hospital of Athens from 1/2018 to 12/2023. Pediatric patients (0–16 years) diagnosed with mastoiditis, orbital cellulitis, and related complications were included. Data were collected across three periods: pre-pandemic (1/2018–3/2020), during the pandemic (4/2020–6/2021), and post-pandemic (7/2021–12/2023). Statistical analyses compared demographic, clinical, and microbiological characteristics between the periods. A total of 176 cases were included (76 mastoiditis and 100 orbital cellulitis cases). The in-hospital incidence of both infections increased significantly post-pandemic compared to the period before the pandemic (mastoiditis: 5.5 vs.13.6 per 1000 admissions; orbital cellulitis: 4.8 vs. 21.8 per 1000 admissions, *P* < 0.001). *Streptococcus* and *Staphylococcus* species predominated across the three periods. Median (IQR) age was not significantly different between the two periods among patients with mastoiditis (pre-pandemic: 5.6 (3.5) years vs. post-pandemic: 3.3 (3.4) years, *P* = 0.12) and among patients with orbital cellulitis (pre-pandemic: 9.2 (6.6) years vs. post-pandemic: 8 (9.2) years, *P* = 0.50). Regarding the complications, the rate of intracranial empyema/abscess development among patients with orbital cellulitis, but not mastoiditis, was lower post-pandemic compared to pre-pandemic (30% vs. 10.1%, *P* = 0.03).

*Conclusion*: The study findings demonstrate a significant rise in the post-COVID-19 in-hospital incidence of mastoiditis and orbital cellulitis in children. Future epidemiological surveillance of these complications and pathogen-specific dynamics, are important to develop prevention strategies for these severe pediatric infections.

**Table:** 

**What is Known:**
• *The COVID-19 pandemic shifted the epidemiology of severe upper respiratory tract infections.*
• *Data on the epidemiology of severe sinogenic and otogenic infections and their intracranial complications during and after the acute phase of the COVID-19 pandemic are limited in Europe.*
**What is New:**
• *This study highlights a notable increase in the in-hospital incidence of mastoiditis and orbital cellulitis during the post-COVID-19 pandemic era, likely associated with immunity debt and alterations in respiratory microbiota.*
• *Despite the increase in pediatric mastoiditis and orbital cellulitis cases, the rates of complications and surgical management remained stable throughout the study period.*

**Supplementary Information:**

The online version contains supplementary material available at 10.1007/s00431-025-06188-4.

## Introduction

Upper respiratory tract infections (URIs), including acute otitis media, sinusitis, and their complications, mastoiditis and orbital cellulitis, are very common in children [1]. The incidence of mastoiditis in developed countries ranges from 1.2 to 4.2 per 100,000 children per year, whereas the one of orbital cellulitis is estimated around 1.6 per 100,000 children per year [2–4]. Despite the potential for serious complications, a broad-spectrum intravenous antibiotic treatment seems to be an effective treatment [5, 6].

Intracranial infections (IIs), including epidural and intraparenchymal abscesses, epidural and subdural empyemas, are more rare but serious complications of acute otitis media, sinusitis, mastoiditis and orbital cellulitis [7]. Intracranial infections are more common in children and young adults resulting in significant morbidity and mortality [8, 9]. Anaerobic and microaerophilic streptococci, such as the *Streptococcus milleri* group (*Streptococcus anginosus* or *Streptococcus intermedius*) are the main cause of IIs. However, some of these infections are multimicrobial [7, 10]. Treatment typically requires neurosurgical and otolaryngological interventions accompanied by antibiotics. In most cases, early diagnosis and adequate treatment lead to good outcome [11].

Severe Acute Respiratory Syndrome Coronavirus 2 (SARS-CoV-2) caused a global pandemic with significant morbidity and mortality [12]. The pandemic shifted the epidemiology of URIs, including mastoiditis, with a dropping incidence, followed by an increase after the lockdown [13, 14]. However, since the onset of COVID-19 pandemic, the frequency of intracranial pyogenic complications has significantly increased [15]. In 2022, the Center for Disease Control and Prevention (CDC) noted an increase of intracranial complications in the USA. During 2021–2022, more streptococci-related intracranial empyema or abscess were reported. Another study in the USA, also, reported a 236% increase of infectious intracranial complications [16, 17].

Data on the epidemiology of severe sinogenic and otogenic infections and their intracranial complications during and after the acute phase of the COVID-19 pandemic are limited in Europe. The purpose of this study is to evaluate the impact of COVID-19 pandemic on epidemiology and characteristics of severe infections, such as mastoiditis and orbital cellulitis and their complications.

## Materials and methods

A retrospective observational study was carried out at “Aghia Sophia” Children’s Hospital in Athens, from January 1 st, 2018, to December 31 st, 2023. This is the largest tertiary pediatric hospital in Greece (750 beds), serving approximately 40% of the pediatric population in the Athens metropolitan area and severe cases of regional hospitals.

The study population consisted of all children aged 0–16 years old, who were hospitalized with mastoiditis, orbital cellulitis and otogenic or sinogenic intracranial complications at “Aghia Sophia” Children’s General Hospital in Athens (Neurosurgery and Otorhinolaryngology departments) during the study period.

Mastoiditis, orbital cellulitis cases and their complications were identified by search for all the relevant ICD-10 (International Classification of Diseases) codes. More specifically, mastoiditis cases were identified by Η70.0, H70.1, H70.8, H70.9, H70, H74, H74.8, H74.9, H95 and orbital cellulitis cases by H05, H05.0, H05.8. Data from patients’ records, including patients’ demographic information, medical history, risk factors, clinical presentation, surgical management, microbiology results, antibiotic treatment, duration of the treatment, complications, and outcomes, were also recorded. Patients with chronic sinusitis–otitis–mastoiditis and the presence of cerebrospinal fluid (CSF) shunt-related infections were excluded.

In Greece, the first COVID-19 patient was diagnosed on 26 February 2020 and educational institutions were closed in March 2020. Stricter measures were implemented, leading to a general lockdown on 23 March 2020. Following a second wave of the pandemic, schools eventually reopened in April 2021 [18]. Based on the timeline of lockdown restrictions and school closures, the study period was divided into three subperiods: before (January 2018–March 2020, period 1), during (April 2020–June 2021, period 2), and after (July 2021–December 2023, period 3) the COVID-19 lockdown. In order to calculate the in-hospital incidence of mastoiditis and orbital cellulitis, hospital admission data by department (Neurosurgery and Otorhinolaryngology departments) were collected.

The study was approved by the institution’s Ethical and Research Committee (approval protocol number: 2936–02/02/24) and all procedures were conducted in accordance with the 1964 Declaration of Helsinki and its later amendments.

For statistical analysis, *x*^2^ test or Fisher’s exact test was used to compare data between the study subperiods. An analysis with *t*-test was conducted to compare the continuous data between the three periods. Statistical significance was set at 0.05. Statistical analysis was conducted using SAS (v 9.4) statistical analysis software.

## Results

A total of 176 pediatric patients with a median age of 5.65 years old (range: 0.5–15.7 years) with the diagnosis of mastoiditis or orbital cellulitis were enrolled in the study across the three different periods; 43 patients during the pre-pandemic period (Period 1: January 2018–March 2020), 21 during the COVID-19 pandemic (Period 2: April 2020–June 2021), and 112 during the post-pandemic period (Period 3: July 2021–December 2023).

**Fig. 1:**
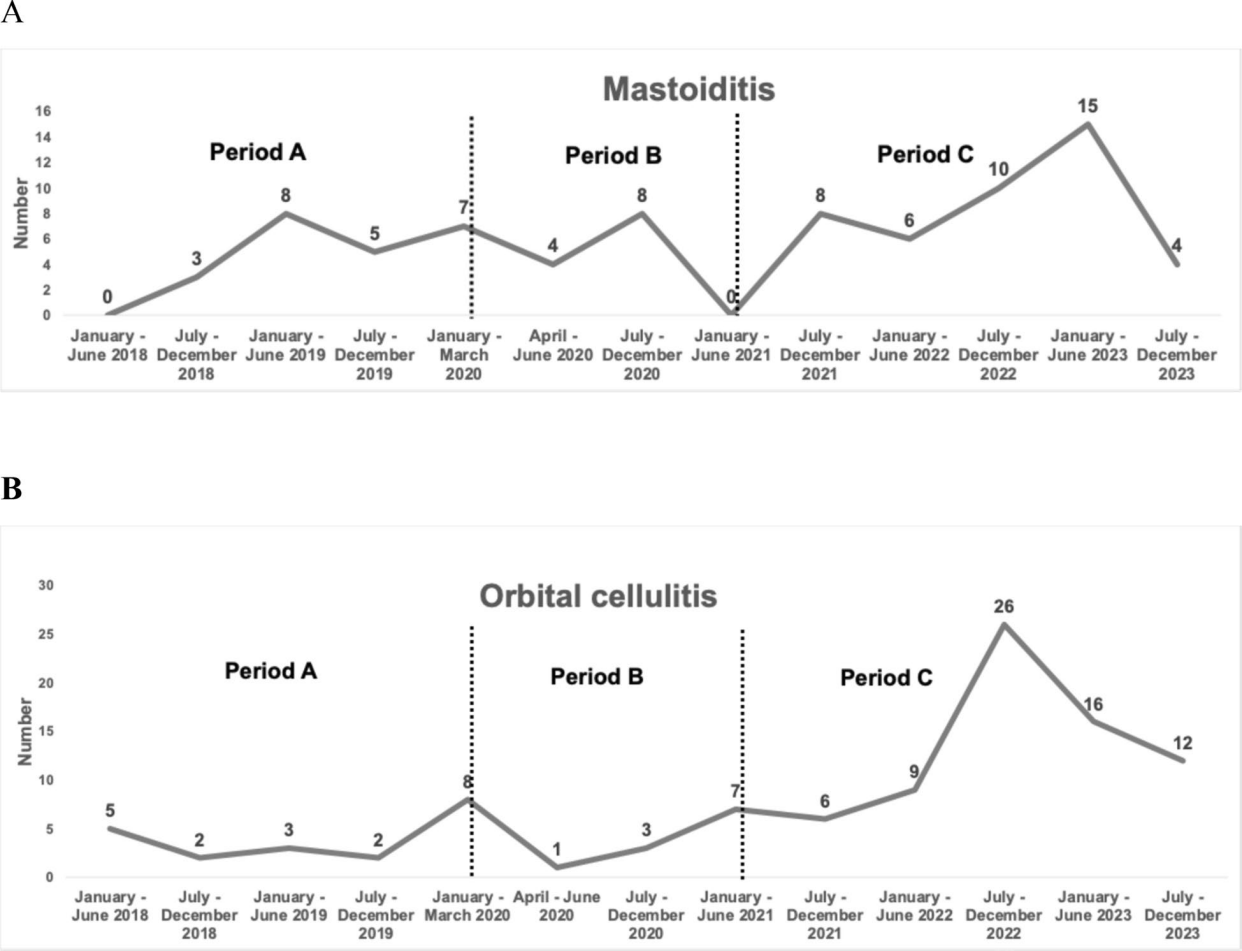
**Α** Number of patients with mastoiditis during the 3 study periods [Period 1 (pre-pandemic): January 2018–March 2020, Period 2 (during pandemic): April 2020–June 2021, and Period 3 (post-pandemic): July 2021–December 2023], **Β** Number of patients with orbital cellulitis during the 3 study periods [Period 1 (pre-pandemic): January 2018–March 2020, Period 2 (during pandemic): April 2020–June 2021, and Period 3 (post-pandemic): July 2021–December 2023]

During the study period, 76 cases of mastoiditis [Period 1: 23 cases (30.3%); Period 2: 10 cases (13.1%); Period 3: 43 cases (56.6%)] and 100 cases of orbital cellulitis [Period 1: 20 cases (20%); Period 2: 11 cases (11%); Period 3: 69 cases (69%)] were identified. A more detailed semester-based trend of mastoiditis and orbital cellulitis cases (N) over the six-year study period is presented in Fig. 1A and B. The majority of these cases were managed in the Otorhinolaryngology Department, which accounted for 70 mastoiditis and 98 orbital cellulitis cases. Comparing the in-hospital incidence of mastoiditis cases across the three periods, statistically significant difference was detected (*P* < 0.001) (Table 1). The same trend was observed in the in-hospital incidence of orbital cellulitis cases across the three periods (*P* < 0.001) (Table 1).

**Table 1:** Distribution of diagnosed cases of mastoiditis (N = 76) and orbital cellulitis (N = 100) admitted to the Neurosurgery and Otorhinolaryngology Departments during the 3 study periods [Period 1 (pre-pandemic): January 2018–March 2020, Period 2 (during pandemic): April 2020–June 2021, and Period 3 (post-pandemic): July 2021–December 2023]

Periods	Admissions N (%)	Mastoiditis			Orbital cellulitis		
		Ν (%)	‰ of hospital admissions	*P*-value	Ν (%)	‰ of hospital admissions	*P*-value
Period1 (pre-pandemic)	4152 (45.7)	23 (30.3)	5.5	** < 0.001**	20 (20.0)	4.8	** < 0.001**
Period 2 (during pandemic)	1762 (19.4)	10 (13.1)	5.7		11 (11.0)	6.2	
Period 3 (post-pandemic)	3165 (34.9)	43 (56.6)	13.6		69 (69.0)	21.8	
Total	9079 (100.0)	76 (100.0)	8.3		100 (100.0)	11.0	

**Fig. 2:**
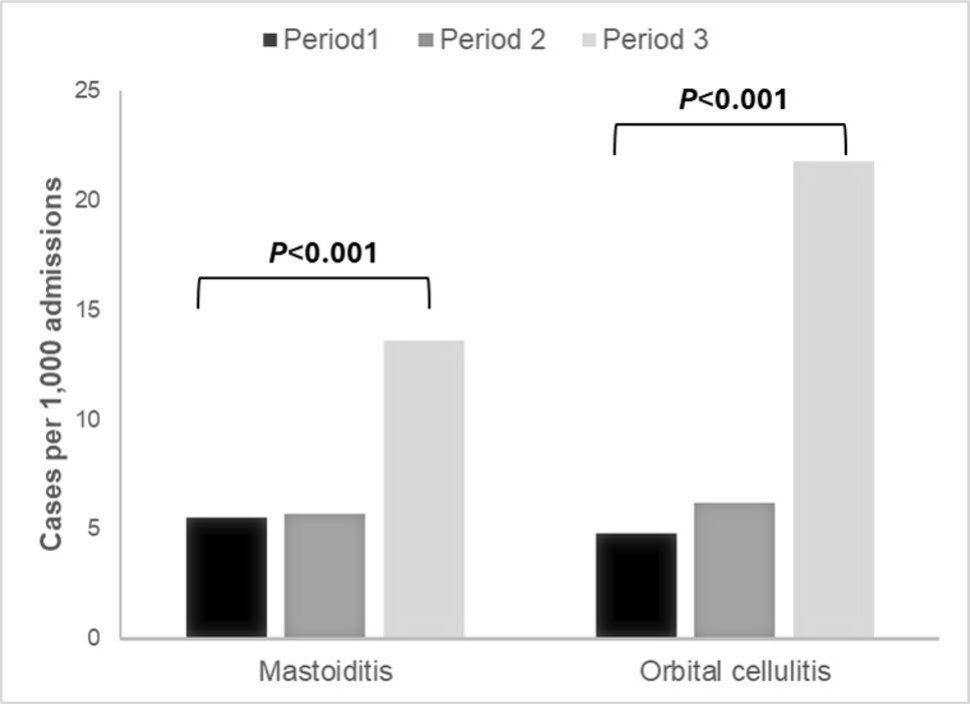
Distribution of the in-hospital incidence of cases with mastoiditis and orbital cellulitis (cases per 1000 hospital admissions) during the 3 study periods [Period 1 (pre-pandemic): January 2018–March 2020, Period 2 (during pandemic): April 2020–June 2021, and Period 3 (post-pandemic): July 2021–December 2023]

The in-hospital incidence of mastoiditis cases rose significantly during the post-pandemic period, from a rate of 5.5 per 1000 hospital admissions pre-COVID-19 pandemic compared to 13.6 per 1000 hospital admissions in the post-pandemic period (*P* < 0.001) (Table 1, Fig. 2). In addition, cases with orbital cellulitis increased significantly pre-pandemic from 4.8 per 1000 hospital admissions to 21.8 per 1000 hospital admissions in the post-pandemic period (*P* < 0.001) (Table 1, Fig. 2). The in-hospital incidence of mastoiditis and orbital cellulitis cases was low during the pandemic (5.7 and 6.2 per 1000 hospital admissions, respectively) (Table 1, Fig. 2). A more detailed figure of in-hospital incidence of mastoiditis and orbital cellulitis over the six-year study period is presented in Supplementary Fig. S1.

Demographics and clinical characteristics of the patients diagnosed with mastoiditis during the pre- and post-pandemic period are described in the Table 2. A higher proportion of male patients was noted in the post-pandemic period (74.4%) compared to pre-pandemic (34.8%, *P* = 0.002). Age was not significantly different between the two periods (Median (IQR) pre-pandemic: 5.6 (3.5) years vs. post-pandemic: 3.3 (3.4) years, *P* = 0.12).

**Table 2:** Demographics and clinical characteristics of patients with mastoiditis (N = 66) during the pre- (January 2018–March 2020) and post-pandemic period (July 2021–December 2023)

Characteristics Ν = 66	Pre-pandemic period (N = 23) N (%)	Post-pandemic period (N = 43) N (%)	*P*-value
Sex (male/female)	8/15 (34.8/65.2)	32/11 (74.4/25.6)	**0.002**
Age [median (IQR)] (years)	5.6 (3.5)	3.3 (3.4)	0.12
Previous antimicrobial administration	11(47.8)	17 (39.5)	0.52
Laboratory investigations			
WBC [mean (SD)] (/mm^3^) Neutrophils [mean (SD)] (/mm^3^) Lymphocytes [mean (SD)] (/mm^3^) CRP [mean (SD)] (mg/L)	14,029 (8943) 9817 (8165) 2850 (1585) 106.6 (89.2)	16,949 (6277) 10,702 (4193) 3867 (1956) 97.4 (68.4)	0.13 0.63 **0.04** 0.64
Children on antibiotic treatment before purulent sample	9 (39.1)	14 (32.5)	0.6
Positive pus culture	5 (21.7)	20 (46.5)	**0.048**
Complications (total)	8 (34.8)	12 (27.9)	0.43
Cerebral venous sinus thrombosis Intracranial empyema/abscess Facial nerve palsy	4 (17.4) 3 (13.0) 1 (4.3)	6 (13.9) 4 (9.3) 2 (4.6)	0.71 0.69 0.95
Surgery	20 (87.0)	34 (79.1)	0.52
Duration of hospitalization (days) [mean (SD)]	11.7 (5.8)	10.6 (3.8)	0.40

Also, there was no statistically significant difference in the previous antibiotic use among patients with mastoiditis (47.8% pre-pandemic vs. 39.5% post-pandemic, *P* = 0.52). The laboratory findings between the pre- and post-pandemic periods, such as white blood cell count (WBC) and C-reactive protein (CRP) levels were not statistically significantly different.

Regarding complications, including mainly cerebral venous sinus thrombosis and intracranial abscesses, there was no significant difference between pre-pandemic (8/23, 34.8%) and post-pandemic period (11/43, 25.6%) among patients with mastoiditis (*P* = 0.43). Surgical intervention was required in 79.1% of mastoiditis cases post-pandemic, with no statistically significant difference in the rates across periods (87.0% pre-pandemic vs. 79.1% post-pandemic, *P* = 0.52). The average duration of hospitalization for mastoiditis decreased from 11.7 days pre-pandemic to 10.6 days post-pandemic (*P* = 0.40).

Demographics and clinical characteristics of the patients diagnosed with orbital cellulitis during the pre- and post-pandemic period are described in the Table 3. During the pre-pandemic period, 70% of cases were males compared to 56.5% during the post-pandemic period (*P* = 0.28). Median (IQR) age was not significantly different between the two periods (pre-pandemic: 9.2 (6.6) years vs. post-pandemic: 8 (9.2) years, *P* = 0.50).

**Table 3:** Demographics and clinical characteristics of patients with orbital cellulitis (N = 89) during the pre- (January 2018–March 2020) and post-pandemic period (July 2021–December 2023)

Characteristics Ν = 89	Pre-pandemic period (N = 20) N (%)	Post-pandemic period (N = 69) N (%)	*P*-value
Sex (male/female)	14/6 (70.0/30.0)	39/30 (56.5/43.5)	0.28
Age [median (IQR)] (years)	9.2 (6.6)	8 (9.2)	0.50
Previous antimicrobial administration	8(40.0)	23 (33.3)	0.58
Laboratory investigations			
WBC [mean (SD)] (/mm^3^) Neutrophils [mean (SD)] (/mm^3^) Lymphocytes [mean (SD)] (/mm^3^) CRP [mean (SD)] (mg/L)	13,559 (5514) 8782 (4210) 3093 (2935) 83.6 (105.9)	14,161 (5440) 9580 (5117) 2977 (2224) 63.3 (50.8)	0.67 0.53 0.85 0.41
Children on antibiotic treatment before purulent sample	2 (10)	3 (4.4)	0.31
Positive pus culture	4 (20.0)	4 (5.8)	0.07
Complications (total)	6 (30.0)	9 (13.0)	0.09
Intracranial empyema/abscess Optic neuritis Pott’s tumor	6 (30.0) 0 0	7 (10.1) 1 (1.4) 1 (1.4)	**0.03** 0.59 0.59
Surgery	6 (30.0)	9 (13.0)	0.09
Duration of hospitalization (days) [mean (SD)]	9.8 (7.1)	8.1 (3.9)	0.29

There was no statistically significant difference in the previous antibiotic use among patients with orbital cellulitis (40% pre-pandemic vs. 33.3% post-pandemic, *P* = 0.58). Also, there was no significant difference in the laboratory findings between the two different periods, such as WBC and CRP levels (mean (SD) WBC: 13,559 (5514)/mm^3^ pre-pandemic vs. 14,161 (5440)/mm^3^ post-pandemic, *P* = 0.67 and mean (SD) CRP: 83.6 (105.9) mg/L pre-pandemic vs. 63.3 (50.8) mg/L post-pandemic, *P* = 0.41).

Regarding the complications rates, they were higher in the pre-pandemic period, however, there were not statistically significant differences, with the exception of intracranial empyemas/abscesses development among patients with orbital cellulitis, which were lower post-pandemic compared to pre-pandemic (6/20, 30% vs. 8/69, 10.1%, *P* = 0.03). Surgical intervention was performed in 13% of post-pandemic cases, which is lower than the pre-pandemic rate of 30% (*P* = 0.09). Of note, there were no significant age differences who underwent surgical intervention post-pandemic compared to pre-pandemic. Specifically, the mean age of children requiring surgery during the post-pandemic period was 6.1 years (SD: 4.75) compared to 7.8 years during the pre-pandemic (SD: 4.08) (*P* = 0.12). Finally, hospitalization duration was comparable, with mean durations of 8.1 days post-pandemic and 9.8 days pre-pandemic (*P* = 0.29).

In patients with mastoiditis, positive pus culture results increased significantly after the pandemic, from 21.7% pre-pandemic to 46.5% post-pandemic (*P* = 0.048) (Table 2). Regarding the pathogens isolated from the pus cultures obtained from the patients with mastoiditis and orbital cellulitis during the three periods are reported in the Table 4. *Staphylococcus* spp. and *Streptococcus* spp. were the most frequently isolated pathogens in patients with mastoiditis, with an increase in polymicrobial infections, including anaerobic and gram-negative bacteria during the post-pandemic period (Table 4). In patients with orbital cellulitis, a decrease in positive pus cultures was observed post-pandemic (20% pre-pandemic vs. 5.8% post-pandemic, *P* = 0.07) (Table 3). Patients with orbital cellulitis displayed higher polymicrobial infections post-pandemic, primarily involving *Staphylococcus* spp. and *Streptococcus* spp. in combination with anaerobic and gram-negative bacteria (Table 4).

**Table 4:** Pathogens isolated from the pus cultures obtained from the patients with orbital cellulitis (N = 8) and mastoiditis (N = 25) during the pre- (January 2018–March 2020) and post-pandemic period (July 2021–December 2023)

Isolated pathogens	Orbital cellulitis		Mastoiditis	
	Pre-pandemic period (N = 4) N (%)	Post-pandemic period (N = 4) N (%)	Pre-pandemic period (N = 5) N (%)	Post-pandemic period (N = 20) N (%)
Monomicrobial infection	3 (75)	1 (25)	4 (80)	11(55)
Polymicrobial infection	1(25)	3 (75)	1(20)	9 (45)
*Streptococcus anginosus*, *Streptococcus mitis*, Coagulase negative *Staphylococcus*, Gram (+) anaerobic rods	N/A	N/A	0	1 (11.1)
*Streptococcus anginosus*, Coagulase negative *Staphylococcus*, Gram (+) anaerobic rods	N/A	N/A	0	1 (11.1)
*Streptococcus pyogenes*, Coagulase negative *Staphylococcus*, Gram (+) anaerobic rods	N/A	N/A	0	2 (22.2)
Coagulase negative *Staphylococcus*, Gram (−) anaerobic rods	N/A	N/A	0	1 (11.1)
*Streptococcus pyogenes*, Coagulase negative *Staphylococcus*	1 (100)	0	0	1 (11.1)
*Streptococcus pneumoniae*, Coagulase negative *Staphylococcus*	N/A	N/A	1 (100)	1 (11.1)
*Streptococcus pneumoniae*, *Haemophilus* spp.	N/A	N/A	0	1 (11.1)
*Pseudomonas* spp. Gram (− anaerobic rods	N/A	N/A	0	1 (11.1)
*Klebsiella* spp. Gram (+) anaerobic rods, Gram (−) anaerobic cocci	0	1 (33.3)	N/A	N/A
*Streptococcus anginosus*, *Streptococcus pneumoniae*, Gram (+) anaerobic cocci	0	1 (33.3)	N/A	N/A
*Streptococcus mitis*, Coagulase negative *Staphylococcus*,* Micrococcus luteus*	0	1 (33.3)	N/A	N/A
Type of microorganism				
*Staphylococcus aureus*	0	1 (25)	0	3 (15)
Coagulase negative* Staphylococcus*	3 (75)	1 (25)	2 (40)	10 (50)
*Streptococcus pneumoniae*	0	1 (25)	1 (20)	2 (10)
*Streptococcus anginosus group*	0	1 (25)	0	3 (15)
*Streptococcus mitis*	0	1 (25)	1 (20)	1 (5)
*Streptococcus pyogenes*	1 (25)	0	1 (20)	3 (15)
Gram (+) anaerobic rods	0	1 (25)	0	5 (25)
Gram (+) anaerobic cocci	1 (25)	1 (25)	N/A	N/A
Gram (−) anaerobic rods	N/A	N/A	0	1 (5)
Gram (−) anaerobic cocci	0	1 (25)	N/A	N/A
*Klebsiella* spp.	0	1 (25)	N/A	N/A
*Micrococcus luteus*	0	1 (25)	N/A	N/A
*Pseudomonas* spp.	N/A	N/A	1 (20)	1 (5)
*Haemophilus* spp.	N/A	N/A	0	1 (5)
*Enterococcus* spp.	N/A	N/A	0	2 (10)

## Discussion

In the present study, we evaluated the impact of COVID-19 pandemic on the epidemiology and characteristics of mastoiditis and orbital cellulitis and their complications in children. A significant increase in the in-hospital incidence of mastoiditis and orbital cellulitis cases during the post-pandemic period was observed compared to the period before and during the pandemic. Indeed, a study from Germany showed a rise in the acute mastoiditis cases in children aged mainly 1–5 years old at the end of COVID-19 pandemic after the reduction of non-pharmaceutical interventions (NPI), compared to pre-pandemic period (2011–2019) [19]. In addition, another study from Germany showed a tenfold increase in acute pediatric mastoiditis after COVID-19 restrictions [20].

Several studies showed a decrease of the total pediatric admissions related to the head and neck infections, including mastoiditis, sinusitis and orbital cellulitis, following the implementation of COVID-19 restrictions, with no significant difference in mean age patient, admission duration and surgical rates [21]. Furthermore, a study involving 8 pediatric hospitals of the USA from March 2020 to March 2022 observed a decrease of orbital cellulitis, sinusitis and mastoiditis by 14.5%, 31.9% and 24.7%, respectively [22]. A national observational study in the UK showed a decreased incidence in pediatric acute mastoiditis during the post-COVID-19 period (December 2020–February 2021) in comparison to the pre-COVID-19 period, possibly due to the loss of upper respiratory tract infections driven winter peak in infection [14]. However, this reduction in the cases of mastoiditis and orbital cellulitis may be due to the fact that these studies were conducted during the implementation of COVID-19 restrictions and the early period after the removal of these measures.

In the present study, the total complication rates were higher in the pre-pandemic period, however, there were not statistically significant differences, with the exception of intracranial abscesses and empyemas following orbital cellulitis, but not mastoiditis, which decreased during the post-COVID-19 compared to the pre-pandemic period. In addition, the rate of intracranial empyemas was 9.3% and 10.1% in the children with mastoiditis and orbital cellulitis during the post-pandemic period, which are in line with previous studies ranging from 4 to 16% during the pre-COVID-19 era [23, 24]. Similarly, one study by Goldberg-Bockhorn et al. demonstrated a complication rate of 13% in children with mastoiditis and a trend toward a slow increase of complications during the post-pandemic period, but this change was not significant [19]. In contrast, another study from the USA showed that mastoiditis complicated by intracranial abscess decreased by 116.7% [22]. In addition, several studies have reported a notable increase in the epidemiology of pediatric sinogenic and otogenic intracranial complications during and after the COVID-19 pandemic, which may be related to the indirect effects of COVID-19 [10, 16, 25, 26].

Also, it has been shown a slight rise in the incidence of septic cerebral venous sinus thrombosis in the setting of pediatric sinogenic and otogenic intracranial infections during the COVID-19 pandemic [27]. Our findings revealed that there was no significant difference in surgery rates and duration of hospitalization pre- and post-COVID-19 era, which are in line with other studies, which indicated a decrease in surgeries among pediatric patients with acute mastoiditis or other complicated upper respiratory infections during the pandemic, but during the subsequent years after the pandemic, the surgery rate reached to pre-pandemic levels as well as a similar morbidity and mortality rate between the different periods [14, 19, 26]. Several possible associations between the COVID-19 pandemic and the complicated infections of the upper respiratory infections have been described [25]. COVID-19 infection has been associated with a range of immunological complications and various secondary bacterial infections [28].

Non-pharmaceutical interventions such as social distancing, universal mask-wearing, and implementation of lockdown measures were implied in the onset of the pandemic, leading to a phenomenon called “immunity debt” [29]. According to this theory, the lack of active immunization against common endemic pathogens for an extended period due to non-exposure, leads to reduced immune memory. This may result in an increased frequency and severity of infections globally, such as the case of respiratory syncytial virus [29–31]. Whether the “immunity debt” caused this sudden increase in the frequency of paranasal sinusitis and other infections is yet to be confirmed. Other theory proposes the disruption of the respiratory microbiota and replacement with more virulent bacterial species, as well as the immune dysregulation and mucosal barrier dysfunction may be some of the mechanisms that can result in severe complicated infections of the upper respiratory infections, such as mastoiditis and orbital cellulitis in children [25].

Regarding pathogen identification, recent reports presented a notable increase of *Streptococcus intermedius* and other *Streptococcus anginosus* group species among the pediatric patients with sinogenic or otogenic intracranial purulent infections after the COVID-19 pandemic [15]. More specifically, in the USA, after a decline in cases with streptococcal intracranial infections at the onset of the COVID-19 pandemic, cases increased and peaked in March 2022 and then declined to baseline levels. *S. imtermedius* was the most prevalent isolated microorganism, followed by *S. anginosus* and *S. constellatus* [17]. Thus, clinical presentation and microbiological features were stable during the pre- and post-COVID-19 era, and there was no evidence of increased case severity, increased antimicrobial resistance or genetic relatedness of streptococcal isolates [17]. Another study showed a rise in pediatric sinusitis or otitis related intracranial infections, with *S. imtermedius*, *S. anginosus* and *S. constellatus* being more prevalent isolated microorganisms [25]. An additional study demonstrated that among sinogenic abscesses in children, *S. imtermedius* was the most prevalent, while among otogenic ones, *S. pyogenes* was the most common microorganism [32]. However, one study conducted before the onset of pandemic reported that the incidence of *S. anginosus*-related infections and particularly, complicated sinusitis increased 12% per year from 2010 to 2016 [33].

Therefore, the increased cases of *S. anginosus* group species infections in children after the pandemic may be due to seasonal fluctuations and a redistribution of cases over time and may result from changes in antimicrobial prescribing practices and in nasopharyngeal colonization due to childhood vaccinations, rather than to the effects of the COVID-19 pandemic only. Two studies from Germany evaluating acute mastoiditis in children before, during and after COVID-19 restrictions noted that there were no significant differences in pathogen distribution between the periods, with *S. pneumoniae* and *S. pyogenes* being the most commonly isolated microorganisms [19, 20]. In our study, *Streptococcus* spp. and *Staphylococcus* spp. were more frequently isolated pathogens, with no significant difference between the study periods. However, several purulent samples were obtained after the administration of antimicrobials, which could have affected culture results and identifications of specific bacteria. Also, coagulase negative *Staphyloccoci* (CoNS) are often considered contaminants, particularly in cases involving non-sterile sites or inappropriate specimen collection techniques and the potential role of CoNS identification in clinical samples should be interpreted with caution.

Τhis study has certain limitations, as this is a single center retrospective study and some data, such as the vaccination status of children, were not available. Detailed information regarding the specific types of surgical procedures performed was not available. Furthermore, the findings may have been impacted by changes in healthcare professionals’ practices and clinical experience during the study period and therefore, decision-making and criteria applied for surgical management may be different across the three periods. Additionally, the observed decline in the total number of hospital admissions during the post-pandemic period may reflect system-level changes in healthcare delivery and patient referral patterns and may limit the generalizability of the results. Finally, PCR analysis was not performed to the purulent samples in the current study and this may limit the ability to detect certain pathogens, reducing the diagnostic accuracy. However, this is the first report about the impact of COVID-19 on the epidemiology of mastoiditis and orbital cellulitis in the Greek pediatric population. The data covers a long period (pre-, during and post-COVID-19) in the largest children’s hospital of Greece and the referral pediatric center for the majority of cases of central and southern country, which could be representative of the general population.

In conclusion, this study highlights a notable increase in the in-hospital incidence of mastoiditis and orbital cellulitis during the post-COVID-19 pandemic era, likely associated with factors such as immunity debt and alterations in respiratory microbiota. Despite the increase in pediatric mastoiditis and orbital cellulitis cases, the rates of complications and surgical management remained stable throughout the study period. These findings underscore the need to recognize the indirect effects of the pandemic on severe pediatric infectious diseases and their management. Further prospective, multicenter, international studies should focus on longitudinal monitoring of these trends to confirm the long-term impact of COVID-19 on these infections in the pediatric population, in order to provide new insights into these growing epidemiological patterns, evaluate pathogen-specific dynamics and develop new preventive strategies to diminish the burden of these severe pediatric infections and their complications in the post-pandemic era.

## Supplementary Information

Below is the link to the electronic supplementary material.

Supplementary file1 (DOCX 393 KB)

## Data Availability

No datasets were generated or analysed during the current study.

## Abbreviations

CDC: Center for Disease Control and Prevention
CSF: Cerebrospinal fluid
ICD-10: International Classification of Diseases
IIs: Intracranial Infections
NPI: Non-pharmaceutical interventions
SARS-CoV-2: Severe acute respiratory syndrome coronavirus 2
*S. anginosus*: *Streptococcus* *anginosus*
*S. constellatus*: *Streptococcus constellatus*
*S. imtermedius*: *Streptococcus intermedious*
URIs: Upper respiratory tract infections
