# Supplementary material for: Impact of COVID-19 on the epidemiology of severe sinogenic and otogenic infections and their intracranial complications
Source: Eur J Pediatr. 2025 May 24;184(6):359. doi: 10.1007/s00431-025-06188-4 (PMC12101995; doi:10.1007/s00431-025-06188-4)

**Supplementary Figure S1.** In-hospital incidence/1,000 hospitalizations of mastoiditis and orbital cellulitis per semester (January 2018 – December 2023).


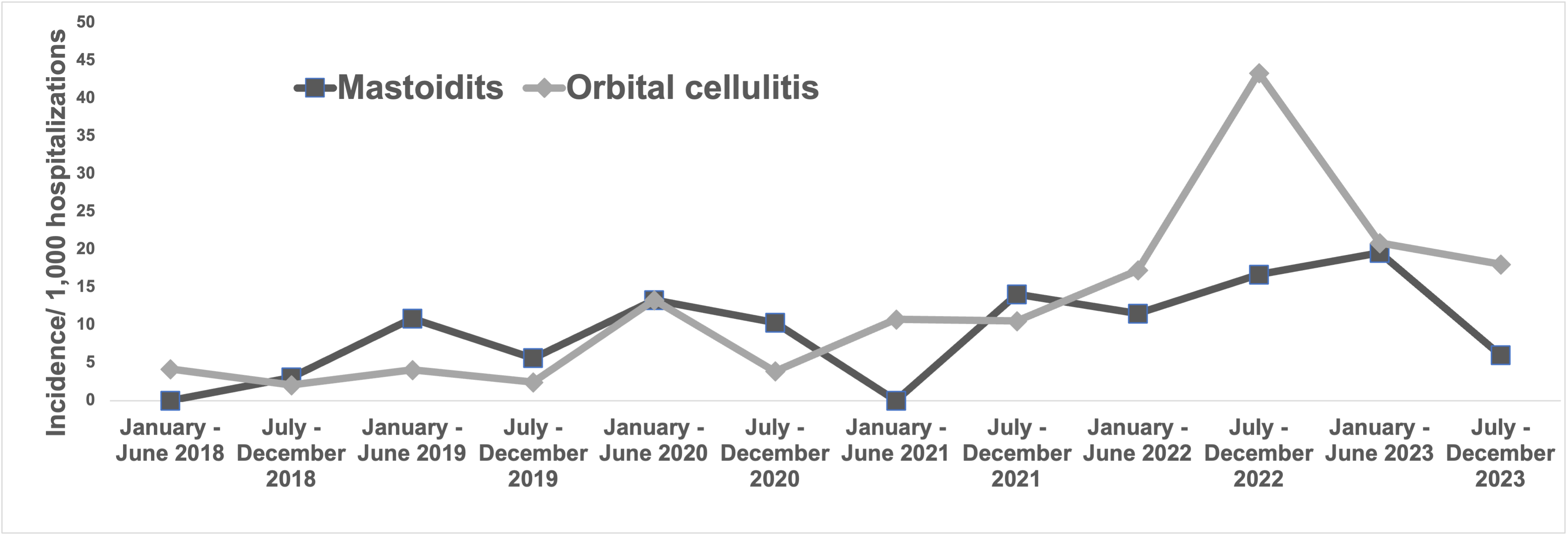

Supplement: Supplementary file 1 — Supplementary file1 (DOCX 393 KB) [file 431_2025_6188_MOESM1_ESM.docx]
